# Uterus preservation is superior to hysterectomy when performing laparoscopic lateral suspension with mesh

**DOI:** 10.1007/s00192-018-3678-3

**Published:** 2018-06-30

**Authors:** Nikolaus Veit-Rubin, Jean Dubuisson, Florin Constantin, Sören Lange, Isabelle Eperon, Victor Gomel, Jean-Bernard Dubuisson

**Affiliations:** 1 Department of Obstetrics and Gynecology, Medical University of Vienna, Währinger Gürtel 18-20, 1090 Vienna, Austria; 2 Faculty of Medicine, University of Geneva, Geneva, Switzerland; 3 Department of Obstetrics and Gynecology, University Hospitals Geneva, Geneva, Switzerland; 4 Department of Obstetrics and Gynecology, Cantonal Hospital of Yverdon-les-Bains, Yverdon-les-Bains, Switzerland; 5 Department of Obstetrics and Gynecology, University of British Columbia, Vancouver, BC Canada; 6 Gynecology Centre, Clinique La Colline, Geneva, Switzerland

**Keywords:** Pelvic organ prolapse, Laparoscopic lateral suspension, Uterine preservation, Patient satisfaction

## Abstract

**Introduction and hypothesis:**

We aimed to compare differences between laparoscopic lateral suspension with mesh (LLS) performed with supracervical hysterectomy (LLSHE) and without hysterectomy (LLSUP).

**Methods:**

We retrospectively collected data from women operated by a single surgeon between 2003 and 2011. From a total of 339 women with symptomatic anterior and/or apical pelvic organ prolapse (POP) and an intact uterus, 224 had LLSUP (70.4%) and 94 had LLSHE (29.6%). Three hundred and sixteen patients were examined at 1 year. Primary outcomes were objective and subjective success at 1 year during clinical evaluation. Secondary outcomes were complications (Clavien-Dindo scale) and mesh exposure. Patient satisfaction was evaluated by telephone interview using a 10-point scale and the Patient Global Impression of Improvement Scale (PGI-I).

**Results:**

LLSUP and LLSHE did not differ for age (mean 57 and 55 years, respectively), preoperative status, complications, and participation at the interview (52 vs 53%). LLSHE is associated with higher mesh exposure (6.5 vs 1.3%, *p* = 0.014) and more frequent use of Mersilene. Titanium-coated and noncoated polypropylene was more frequently used in LLSUP. At 1 year, both anatomic success rate for the anterior compartment (98.7 vs 94.6%, *p* = 0.021) and subjective success rate (83.5 vs 72.8%, *p* = 0.035) were higher for LLSUP. Without hysterectomy, patients more often improved (90.5 vs 76.5%, *p* = 0.013) and would more frequently recommend the procedure (94.5 vs 80.4%, *p* = 0.004).

**Conclusions:**

LLS with or without hysterectomy is a safe technique with high patient satisfaction. The uterus-preserving approach appears to result in better anatomic outcome for the anterior compartment, better subjective outcome, and higher patient satisfaction.

**Electronic supplementary material:**

The online version of this article (10.1007/s00192-018-3678-3) contains supplementary material, which is available to authorized users.

## Introduction

The high prevalence of pelvic organ prolapse (POP) is closely associated with the high risk for woman to undergo surgery for such a condition [1–4]. There is an ongoing debate about the ideal surgical approach for POP in general and the apical compartment in particular [5]. Traditionally, in the presence of a uterus, surgical correction of apical prolapse has included hysterectomy, whereas now, it is believed that the uterus plays more of a passive role in the condition’s development [6]. Although POP has a significant impact on a woman’s quality of life (QoL), a radical surgical approach sacrificing an otherwise healthy organ is frequently questioned [1, 7]. For some women, the uterus is part of their sense of identity, and they would opt for uterine preservation for additional reasons, such as the desire to maintain fertility and the belief it affects sexual function [6]. This change in psychological value has led to an increase in uterine-sparing prolapse surgery [7–9]. Laparoscopic sacrocolpopexy and sacrohysteropexy are now considered the gold standards for correcting apical POP [5]. These techniques require dissection at the level of the promontory, which can be challenging, particularly in obese women and when anatomic variations exist. Sacral area lesions can lead to serious neurological, ureteral, or vascular injuries. In up to 50% of patients, lumbar pain has been described following mesh fixation at the sacrum [10]. In our previous publications, we demonstrated that laparoscopic lateral suspension (LLS) with mesh is a safe and feasible alternative. It avoids dissection at the promontory and can be performed with uterine preservation [11, 12]. We also previously identified uterus-preserving LLS as a factor improving patient satisfaction [13]. In the cross-sectional study reported here, we compared outcomes, complications, and prolapse-associated symptoms in women from the same cohort operated with LLS performed with and without hysterectomy.

## Materials and methods

The local Ethics Committee on Clinical Studies of the Geneva University Hospitals approved the study protocol (no. 14-197). This observational cross-sectional study represents a prospective series of consecutive patients treated by LLS for symptomatic POP between January 2004 and October 2011 by a single surgeon (JBD) at the Geneva University Hospital and who completed a standardized clinical follow-up assessment of patients at 1 year postsurgery. We followed recommendations of the International Urogynecology Association (IUGA) for reporting outcome of surgical procedures for POP [14].

### Preoperative assessment and inclusion criteria

Patients were included for surgical treatment with LLS when they presented with prolapse-related symptoms, such as a sensation of a bulge or a lump in the vagina. Patients were offered a supracervical hysterectomy if they desired removal of the uterus in the presence of uterine abnormalities, such as enlarged leiomyomas, adenomyosis, endometrial hyperplasia, or abnormal uterine bleeding. All other women had a uterus-preserving approach. Clinical evaluation of pelvic organ support was assessed by the simplified Pelvic Organ Prolapse Quantification grading system (POP-Q) [15]. When patients presented with clinically reported stress urinary incontinence (SUI), they were offered concomitant surgical treatment by laparoscopic Burch colposuspension or transobturator suburethral tape or expectative management.

### Surgical technique

LLS aims to treat anterior and apical compartment POP. It is performed under general anesthesia with the patient in the Trendelenburg position. We use a central 10-mm umbilical trocar for the camera and three working trocars (all 5 mm or one 10 mm and one 2 5 mm) in the inguinal regions and suprapubically. In a first step, the vesicovaginal space is dissected until the endopelvic fascia is reached. This deep dissection adequately enables the treatment of concomitant cystocele. In a second step, the rectovaginal space is dissected toward the perineal and anorectal junction. For both steps, the dissection plane is exposed with the help of a flat-blade retractor placed in the vagina and manipulated by the assistant. Third, the mesh is introduced in the peritoneal cavity through a 10-mm trocar. The mesh is T-shaped and has a central rectangular part (~4 × 6 cm) and two long lateral side arms. The central part is flattened over the vesicovaginal dissection plane and fixed to the vaginal fascia with absorbable tackers and four to six separated nonabsorbable sutures. In a fourth step, the skin is incised 2 cm above the iliac crest and 4 cm posterior to the anterior superior iliac spine on both sides over 3 mm, and a laparoscopic grasping forceps is introduced through this new incision (Fig. 1). The latter perforates the aponeurosis perpendicularly and stops behind the peritoneum without perforating it. The forceps is pushed toward the round ligament at the level of its lateral peritoneal insertion through retroperitoneal tunnelization under transperitoneal visualization. The side arms of the mesh previously fixed at the vesicovaginal space can now be grasped and pulled backward through the previously formed tunnel with the tension symmetrically adjusted. Retroperitoneal fibrosis provides adequate attachment of the mesh, with lateral tension-free suspension of the central mesh part attached to the vagina. Hysterocele and cystocele are hereby treated. The peritoneum is closed to completely cover the graft and the side arms cut at the level of the skin (Fig. 2). The posterior compartment was treated separately when there was a prolapse beyond the hymen or preoperative constipation. We either placed a posterior polypropylene mesh laparoscopically in the rectovaginal space after deep dissection, or we performed a posterior colporrhaphy though the vaginal route.

The first patients operated in our series had placement of polyethylene meshes (Mersilene®; Ethicon Inc., Somerville, NJ, USA); recently, only macroporous lightweight polypropylene meshes (Gynemesh®; Ethicon) or titanium-coated polypropylene meshes (TiLOOP®; pfm medical ag, Köln, Germany) have been used. Polyethylene was withdrawn from the market during our study because of an increasing number of reports by other surgeons of mesh erosion.

### Postoperative assessment

Systematic postoperative clinical examination and assessment of lower urinary tract symptoms (LUTS), satisfaction, and prolapse-related symptoms was performed at 12 months. Additionally, we searched hospital files for mesh-related complications.

### Outcome measures

The main outcome measures were subjective and objective cure at 1 year and patient satisfaction at a certain point in time since the operation. Anatomic cure was the first primary outcome and defined as POP-Q sites Ba, C, and Bp less than −1 cm [16]. Subjective cure was the second primary outcome and was considered when the patient did not report any prolapse-related symptoms. Patient satisfaction was the third primary outcome and was assessed using the Patient Global Impression of Improvement (PGI-I) and a 10-point visual analog scale (VAS) for global satisfaction during a telephone interview. Highest possible satisfaction was rated 10 and lowest 0. The interview was conducted between 4 and 10 years after surgery [17]. Patients were considered satisfied when they rated satisfaction with at least 8 and when the PGI-I was answered as “improved” or “very improved.” Secondary outcomes were mesh-related complications. We also reported complication rates using the Clavien-Dindo scale [18].

### Statistical analyses

Continuous variables were reported by their mean ± standard deviation (SD). We considered a *p* value of <0.05 to reflect statistical significance. Categorical variables were described by their number and relative proportion. Variables were compared between groups using the chi-square test. We then assessed whether there was a change in prolapse stages pre- and postoperatively in the same patients (POP-Q point Ba, C, and Bp above −1 cm) using McNemar test. Data analysis was performed using SPSS version 24.0.

## Results

Between 2003 and 2011, 243 patients had uterus-preserving laparoscopic lateral suspension (LLSUP), and 96 patients had laparoscopic lateral suspension after supracervical hysterectomy (LLSHE), with the uterus being removed after intra-abdominal morcellation. Demographic data, preoperative procedures, and conditions are summarized in Table 1 and postoperative conditions in Table 2. Preoperative characteristics did not differ between groups, except for body mass index (BMI) (LLSUP 26.05 ± 4.04 vs LLSHE 27.05 ± 3.73, *p* = .036). All patients had significant stage ≥2 POP in at least two of the three compartments (Table 3). Several patients had concomitant surgery for SUI: 144 of 153 SUI patients in the LLSUP group and 46 of 55 in the LLSHE group. There was no difference between groups in regard to technique used for the procedure (Table 3). The postoperative persistent SUI rate was similar between groups, while the de novo SUI rate was significantly higher in the LLSHE group (4.3 vs 1.8%, *p* = .030) (Table 3).

**Table 1:** Demographic data and preoperative condition of patients treated with laparoscopic lateral suspension

	LLSUP *N* = 243	LLSHE *N* = 96	*P * value
Mean age in years (±SD)	57.28 (±12.08)	55.46 (±10.66)	0.198
Mean BMI in kg/m^2^(±SD)	26.05 (± 4.04)	27.05 (± 3.73)	0.036
Mean parity (±SD)	2.24 (± 1.13)	2.08 (±0.87)	0.215
Mean number of vaginal deliveries (±SD)	2.20 (± 1.14)	2.01 (± 0.86)	0.149
Menopausal women, *N* (%)	169 (69.5)	58 (60.4)	0.107
Sexual activity, *N* (%)^a^	124 (60.8)^a^	56 (70.0)^a^	0.147
Pelvic pain, *N* (%)	18 (7.4)	12 (12.5)	0.137
Dyspareunia, *N* (%)^b^	28 (22.6)^b^	14 (25.0)^b^	0.722
Urinary frequency, *N* (%)	71 (29.2)	23 (24.0)	0.330
Urinary urgency, *N* (%)	49 (20.2)	21 (21.9)	0.726
Dysuria, *N* (%)	34 (14.0)	15 (15.6)	0.700
Stress urinary incontinence, *N* (%)	153 (63.0)	55 (57.3)	0.334
Constipation, *N* (%)	57 (23.5)	20 (20.8)	0.603

Bold data indicates statistical significance

*SD* standard deviation,* BMI* body mass index, *LLSHE* laparoscopic lateral suspension with hysterectomy, *LLSUP* laparoscopic lateral suspension with uterine preservation,

^a^Information obtained from 204 patients in the LLSUP group and 80 patients in the LLSHE group

^b^Among sexually active patients (124 in the LLSUP group, 56 in the LLHE group)

**Table 2:** Peroperative procedures and postoperative condition at 1 year of patients treated with laparoscopic lateral suspension

	LLSUP *N* = 243	LLSHE *N* = 96	*P* value
Type of anterior mesh, *N* (%)			<0.001
Polyethylene	73 (30.0)	62 (64.6)	
Polypropylene	142 (58.4)	26 (27.1)	
Titanium-coated polypropylene	28 (11.5)	8 (8.3)	
SUI operation, *N* (%)			0.152
No	99 (40.7)	50 (52.1)	
Laproscopic Burch colposuspension	137 (56.4)	43 (44.8)	
Transobturator suburethral tape	7 (2.9)	3 (3.1)	
Mean operating time in minutes, median (±SD)	189.26 (± 44.62)	237.13 (± 57.56)	<0.001
Postoperative cohort at 1 year on clinical follow-up,* N*	224	92	
LUTS and other symptoms at 1 year			
Prolapse symptoms, *N* (%)	37 (16.5)	25 (27.2)	0.030
Urinary urgency, *N* (%)	11 (4.9)	4 (4.3)	0.831
Urinary frequency, *N* (%)	5 (2.2)	2 (2.2)	0.975
SUI persistant, *N* (%)	1 (0.4)	2 (2.2)	0.058
SUI de novo, *N* (%)	4 (1.8)	4 (4.3)	0.030
Constipation persistant, *N* (%)	7 (3.1)	11 (12.0)	0.004
Constipation de novo, *N* (%)	5 (2.2)	4 (4.3)	0.008
Sexual activity at 1 year, *N* (%)^a^	164 (82.8)	69 (86.3)	0.483
Dyspareunia, *N* (% from 164 sexually active)	8 (4.9)	3 (4.3)	0.862
Mesh exposure or extrusion (MEE), *N* (%)	3 (1.3)	6 (6.5)	0.012
Anterior MEE, *N* (%)	2 (0.9)	5 (5.4)	0.013
Posterior MEE, *N* (%)	1 (0.4)	2 (2.2)	0.150
Postoperative complications (Clavien grade), *N* (%)			0.052
None	218 (89.7)	90 (93.8)	
1	9 (3.7)	1 (1.0)	
2	13 (5.3)	1 (1.0)	
3	3 (1.2)	4 (4.2)	

Bold data indicates statistical significance

*SUI* stress urnary incontinence,* LUTS* lower urinary tract infection, *LLSHE* laparoscopic lateral suspension with hysterectomy, *LLSUP* laparoscopic lateral suspension with uterine preservation

^a^Information from 198 patients in the LLSUP group and 80 in the LLHE group

**Table 3:** Anatomic outcome

	Preoperatively *N*, (%)		Postoperatively 12 months *N*, (%)		*P* value^a^	*P* value^b^	*P* value^c^	*P* value^d^
	LLSUP (*N* = 243)	LLSHE (*N* = 96)	LLSUP (*N* = 224)	LLSHE (*N* = 92)				
POP-Q point Ba ≥-1	243 (96.3)	89 (92.7)	3 (1.3)	5 (5.4)	<0.001	<0.001	0.160	0.035
POP-Q point C ≥-1	171 (70.4)	74 (77.1)	6 (2.7)	4 (4.3)	<0.001	<0.001	0.214	0.441
POP-Q point Bp ≥-1	158 (65.0)	54 (56.3)	11 (4.9)	4 (4.3)	<0.001	<0.001	0.133	0.831

Bold data indicates statistical significance

*POP-Q* Pelvic Organ Prolapse Quantification system, *LLSHE* laparoscopic lateral suspension with hysterectomy, *LLSUP* laparoscopic lateral suspension with uterine preservation

^a^Comparison between LLSUP preoperatively and 12 months postoperatively, *P* value obtained with McNemar test

^b^Comparison between LLSHE preoperatively and LLSHE 12 months postoperatively, *P* value obtained with McNemar test

^c^Comparison between LLSUP and LLSHE preoperatively, *P* value obtained with Pearson’s chi-square test

^d^Comparison between LLSUP and LLSHE 12 months postoperatively, *P* value obtained with Pearson’s chi-square test

The operating time was significantly longer in the LLSHE group in whom a hysterectomy was performed compared with the LLSUP group in whom the uterus was preserved (mean operating time in minutes 237.13 (± 57.56) vs 189.26 (± 44.62), *p* < 0.001). Groups differed significantly in regard to type of mesh material used. More patients in the LLSHE group were treated with a polyethylene mesh than patients in the LLSUP group (64.6% vs 30%).

There was no conversion to laparotomy. Seven patients had major complications rated grade 3 on the Clavien-Dindo scale (Table 3): three in the LLSUP group vs four in the LLSHE group. In the LLSUP group, two patients had a trocar hernia at the level of a 10-mm trocar insertion (usually the left groin area) and one a vaginal hematoma after posterior colporrhaphy, which was surgically drained. In the LLSHE group, two vesical lesions were immediately sutured: one patient had trocar-related subcutaneous granuloma and one required re-implantation of the ureter on day 16 postoperatively after discovery of a ureterovaginal fistula.

POP symptoms were reported by 16.5% of women in the LLSUP and 27.2% in the LLSHE group 1 year after surgery, indicating a significantly different subjective cure rate of 83.5 vs 72.8% (*p* = 0.030) (Table 3). Overall, there were more sexually active women at 1 year after surgery (LLSUP 82.8%; LLSHE 86.3%) (Table 3) than preoperatively (LLSUP 60.8%; LLSHE 70%) (Table 1). Twenty-three patients were lost to clinical follow-up after 1 year, 20 in the LLSUP group and three in the LLSHE group. There was clinically and statistically significant anatomic improvement for all compartments at 1 year postoperatively in both groups, resulting in an objective cure rate of 98.7 and 94.6% for the anterior, 97.3 and 95.7% for the apical, and 95.1 and 95.7% for the posterior compartments, respectively (Table 2). There was a significantly better improvement rate for the anterior compartment in the LLSUP group (*p* = 0.035) (Table 2). Women in the LLSHE group had significantly higher persistent (12.0 vs 3.1%, *p* = .004) and de novo (4.3 vs 2.2%, *p* = .008) constipation rates compared with women in the LLSUP group (Table 3).

Nine patients had mesh-related complications: three (1.3%) in the LLSUP group compared with a significantly higher number of six (6.5%, *p* = 0.012) in the LLSHE group. In the LLSUP group, two (0.9%) patients had exposure or extrusion of the anterior and one patient (0.4%) of the posterior mesh. In the LLSHE group, five (0.9%) patients had exposure or extrusion of the anterior and two patients (2.2%) of the posterior mesh. The mesh-related complication rate for the anterior graft was significantly higher in the LLSHE group (*p* = 0.013) (Table 3).

One hundered and twenty-seven patients with LLSUP (52.3%) and 51 with LLSHE (53.1%) participated in the telephone interview. The mean follow-up period was significantly shorter in the LLSHE group (22.61 vs 24.91 months, *p* < 0.001). One hundred and fifty-one women were not reachable after at least three attempts, and five refused to participate. Five women died during the study period. Significantly more patients in the LLSUP group reported a VAS score of ≥8 (85.8%) compared with those in the LLSHE group (66.7%) (*p* = 0.004). Improvement (improved or very much improved) in their condition was reported by 90.6% of women in the LLSUP group (on the PGI-I scale, which was significantly more frequent than women in the LLSHE group (76.5%) (*p* = 0.013). Those in the LLSUP group would recommend the operation more often than those in the LLSHE group (94.5 vs 80.4%, respectively, *p* = 0.004). Results of the telephone interview are summarized in Table 4.

**Table 4:** Results of telephone interview

	LLSUP *N* = 243	LLSHE *N* = 96	*P* value*
Patients interviewed, *N* (%)	127 (52.3)	51 (53.1)	0.911
Not reachable after at least 3 attempts, *N* (%)	109 (44.9)	42 (43.8)	
Refused to participate	3 (1.2)	2 (2.1)	
Deceased	4 (1.6)	1 (1.0)	
Months between operation and interview (±SD)	81.02 (24.91)	93.60 (22.61)	<0.001
Recommendation to a relative or friend, *N* (%)	120 (94.5)	41 (80.4)	0.004
Overall satisfaction on VAS (dichotomous)			
≥8	109 (85.8)	34 (66.7)	0.004
≤8	18 (14.2)	17 (33.3)	
PGI-I (dichotomous), *N* (%)			
Very bad to unchanged	12 (9.4)	12 (23.5)	0.013
Improved/much improved	115 (90.6)	39 (76.5)	

*SD* standard deviation,* PGI-I* Patients Global Impression of Improvement scale, *LLSHE* laparoscopic lateral suspension with hysterectomy, *LLSUP* laparoscopic lateral suspension with uterine preservation

*Pearson’s chi-square test

## Discussion

Between 31 and 60% of US women presenting for prolapse care would elect to keep their uterus if surgical outcomes were equally efficacious [19]. One reason might be the knowledge among patients about higher exposure rates after mesh augmented prolapse repair when hysterectomy is performed. However, at the time of our study, debates about mesh surgery were still quite uncommon in the broader public perception.

Uterine preservation is a suitable option in women with POP; however, long-term data are limited, and the need for subsequent hysterectomy is unknown [6, 20]. Our large series demonstrated that the technique of LLS performed with or without hysterectomy is feasible and effective in an overweight population, with low postoperative complications within 1 year. Patients in our study shared comparable characteristics and perioperative outcomes with those in large series on sacrocolpopexy and sacral hysteropexy [21–24]. Both our techniques for POP surgery—uterine preserving and not preserving—demonstrated overall objective success rates >90% at 1 year. Similar results have been reported for sacrocolpopexy and sacral hysteropexy in previous studies [5, 6]. In addition to previous results from our series, we directly compared hysterectomy and uterine-preservation approaches. We present new findings that add further substantial information for the benefit of patients in the context of a growing demand in uterine-preserving strategies for treating prolapse. We can only speculate about the reason for the surprising finding of a better outcome for the anterior compartment in the LLSUP group. Maybe isthmic fixation of the mesh provides better anterior traction on the vaginal wall. The subjective cure rate at 1 year was high in the LLSHE group and even higher in the LLSUP group. Overall, our results showed a positive outcome by definition of the composite criteria described by Barber et al. [16].

Only 2.2% of patients in the LLSUP group and 4.3% in the LLSHE group developed de novo constipation: this is less than the published rates for sacropexy [21–23]. Lesions of the superior hypogastric plexus and spondylodiscitis are known complications of sacral fixation, and numerous cases have been described in the literature [25]. The advantage of our technique is it avoids dissection at the level of the promontory and associated risks, which are commonly described in obese women in particular. The higher de novo constipation rate in the LLSHE group may be explained by potential lesions close to the hypogastric nerves during hysterectomy, with crossing fibers coming from the superior hypogastric plexus and going to the pelvic side wall, which are at risk for damage. LLS resulted in low rates of LUTS and seems to preserve normal sexual function, with higher rates of sexually active patients after the surgery than before in both groups at 1 year postoperatively.

Perioperative complications of Clavien-Dindo grade ≥3 occurred as rarely in both cohorts as in reports that used different techniques [26]. Mesh-related complication rates were comparable with those reported for other laparoscopic prosthetic POP repairs [26]. The current reports indicate that sacral colpopexy associated with hysterectomy has a four times higher risk of mesh exposure (grade B) than sacral colpopexy without hysterectomy [6, 27]. The higher rate in our hysterectomy group is probably due to the more frequent use of polyethylene mesh, which has been withdrawn from the market for this precise reason. The difference in type of mesh material used in both groups represents a significant bias in our study. However, we found no exposure with titanium-coated polypropylene, the benefits of which were demonstrated in hernia repair [28, 29].

Our telephone interview revealed a higher satisfaction in patients who had LLS with uterine preservation. These results may be biased by the difference in mean follow-up period between groups, although overall mean follow-up exceeded 6.5 years in both groups. The higher subjective cure rate and satisfaction in LLSHE patients may illustrate the trend of a positive association women tend to have between their self-image and their uterus.

The strengths of this study are its prospective design with regard to the clinical follow-up as a consecutive series of patients operated with a standardized technique by a single surgeon and the satisfying turnout of >50% in telephone interviews, providing a long-term follow-up about patient satisfaction. However, loss to follow-up rates close to 50% may represent a source for bias. Loss to follow-up was partially due to a highly migratory patient population. Limitations were results based on the experience a single surgeon working in single university center and the substantial differences in mesh materials used over the years. Patient satisfaction was assessed on a long-term basis using objective factors measured at standardized time-points following surgery, but there were significant variations in postoperative follow-up times when this information was obtained. Another shortcoming is the heterogeneity in follow-up intervals for objective and subjective outcome (1 year) and patient satisfaction (4–10 years).

## Conclusion

In conclusion, both LLSUP and LLSHE are feasible and safe approaches for POP in sexually active and obese women and may represent a safe alternative to sacrohysteropexy and sacrocolpopexy, respectively. Uterine preservation is associated with higher satisfaction rates, better short-term subjective outcome, and lower rates of postoperative constipation and de novo SUI. However, there were differences in mesh materials used, and only a prospective controlled randomized study may confirm our results. Overall, patient goals and preferences about preservation of the uterus should be carefully considered during surgical planning and when obtaining informed consent. There is a need for further studies evaluating the safety and efficacy of hysteropexy, since there are limited data regarding risks associated with subsequent pregnancy and delivery.

## Electronic supplementary material

Fig.1 Skin entry point for fixation of the lateral mesh arm during laparoscopic lateral suspension (PNG 12743 kb)

High resolution image (TIFF 130 kb)

Fig. 2 Final aspect after peritoneal closure following uterus-preserving laparoscopic lateral suspension with mesh (PNG 8248 kb)

High resolution image (TIFF 678 kb)

## Abbreviations

POP: Pelvic organ prolapse
LLS: Laparoscopic lateral suspension
LLSUP: Uterus-preserving laparoscopic lateral suspension
LLSHE: Laparoscopic lateral suspension with hysterectomy
POP-Q: Pelvic Organ Prolapse Quantification system
PGI-I: Patient Global Impression of Improvement
VAS: Visual analog scale
LUTS: Lower urinary tract symptoms
