# Supplementary figures and images for: Uterus preservation is superior to hysterectomy when performing laparoscopic lateral suspension with mesh
Source: Int Urogynecol J. 2018 Jun 30;30(4):557–64. doi: 10.1007/s00192-018-3678-3 (PMC6450845; doi:10.1007/s00192-018-3678-3)

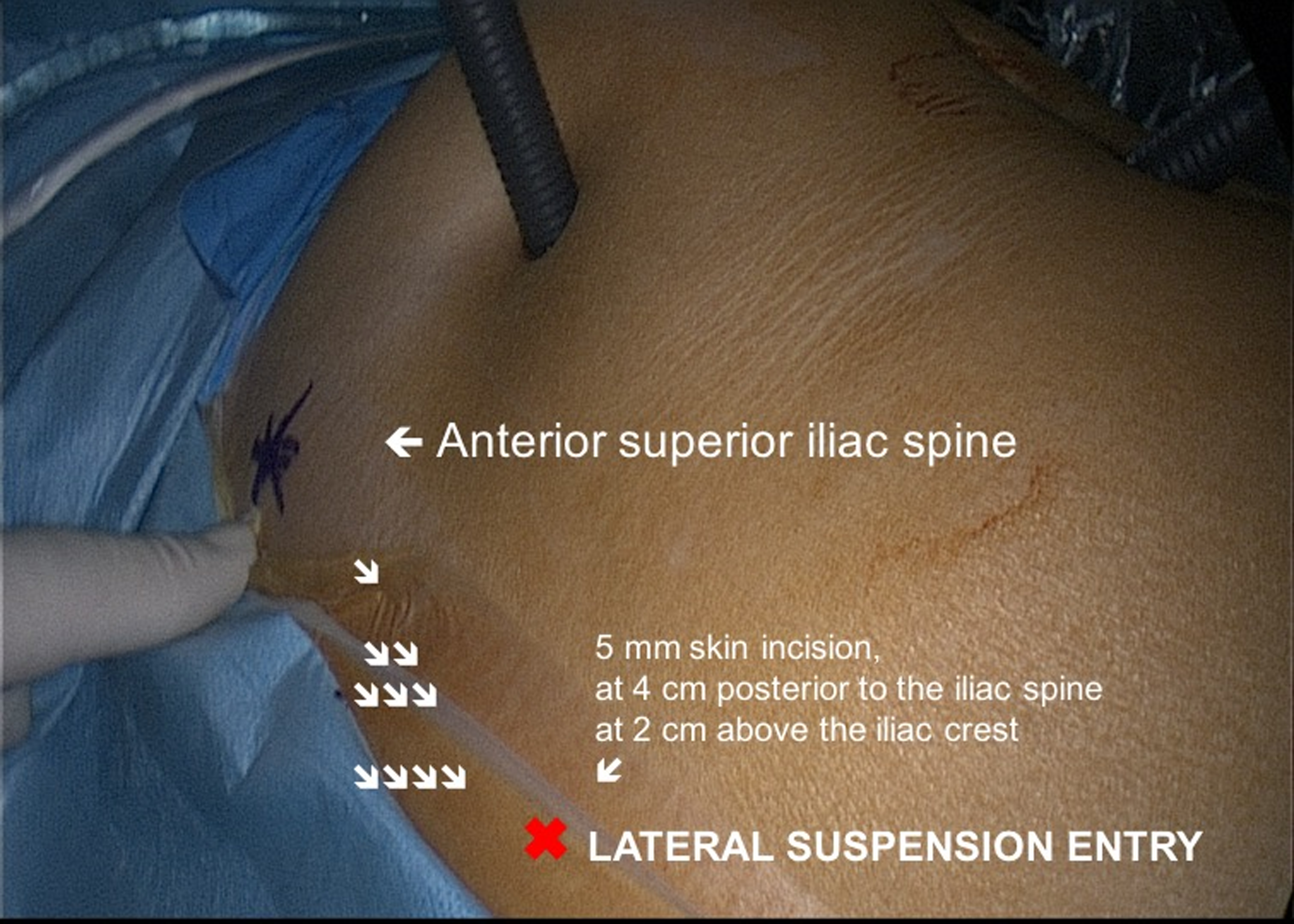

Supplement: Supplementary file 1 — Skin entry point for fixation of the lateral mesh arm during laparoscopic lateral suspension (PNG 12743 kb) [file 192_2018_3678_Fig1_ESM.png]

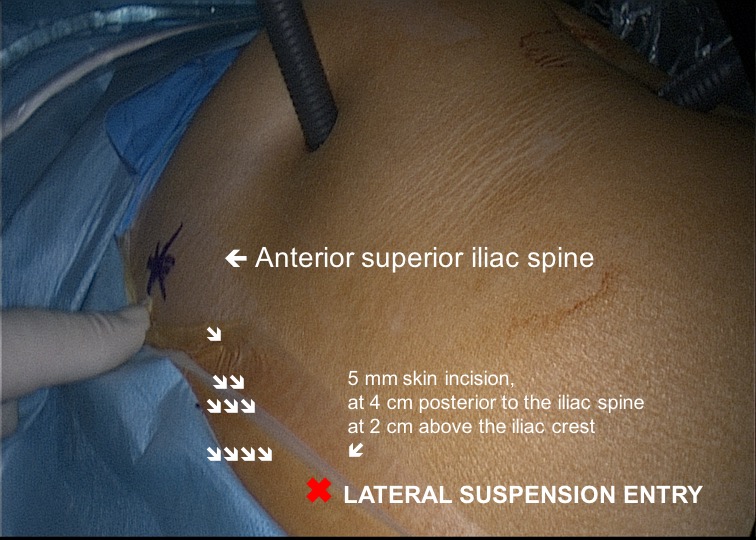

Supplement: Supplementary file 2 — High resolution image (TIFF 130 kb) [file 192_2018_3678_MOESM1_ESM.tiff]

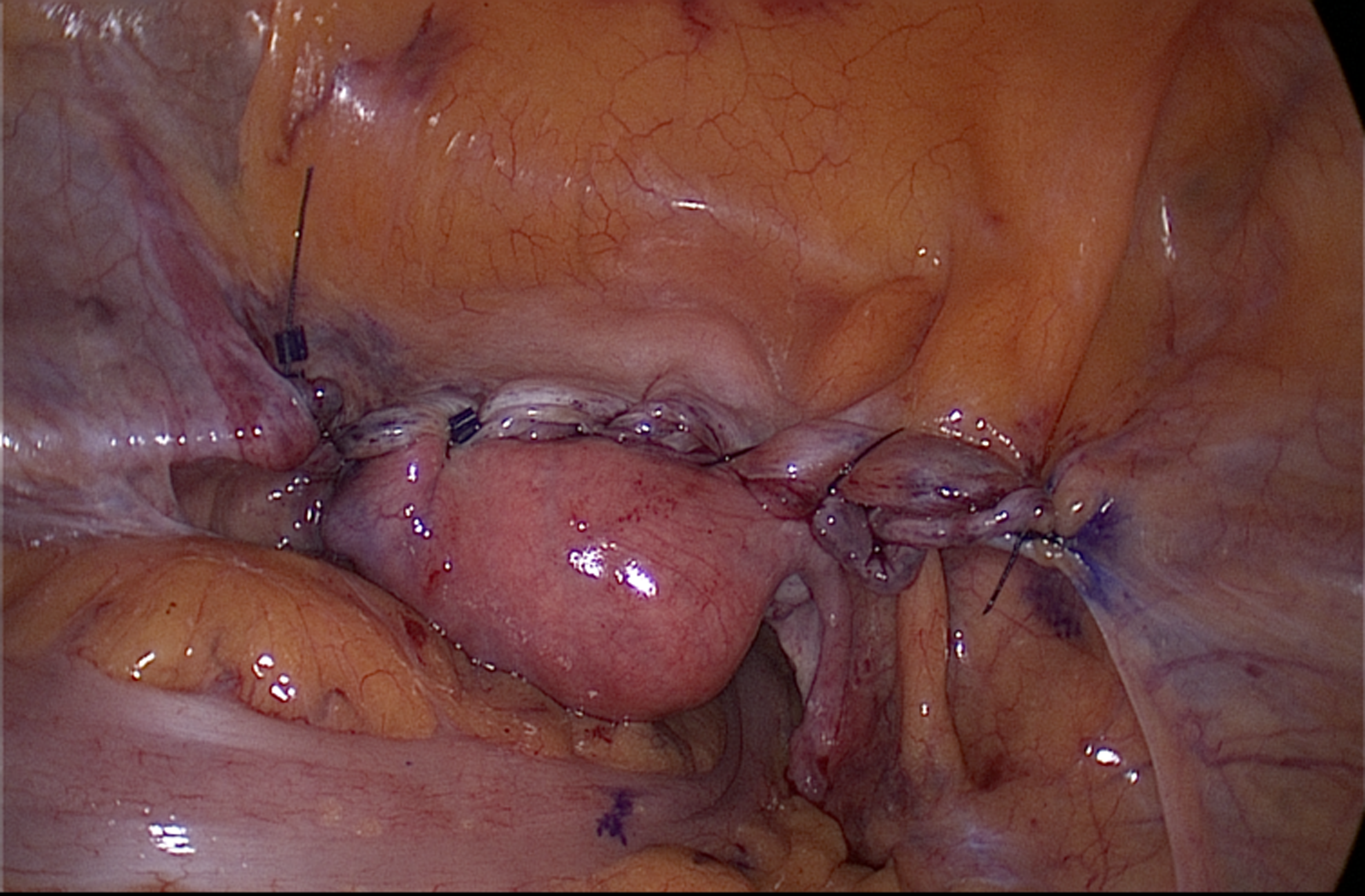

Supplement: Supplementary file 3 — Final aspect after peritoneal closure following uterus-preserving laparoscopic lateral suspension with mesh (PNG 8248 kb) [file 192_2018_3678_Fig2_ESM.png]

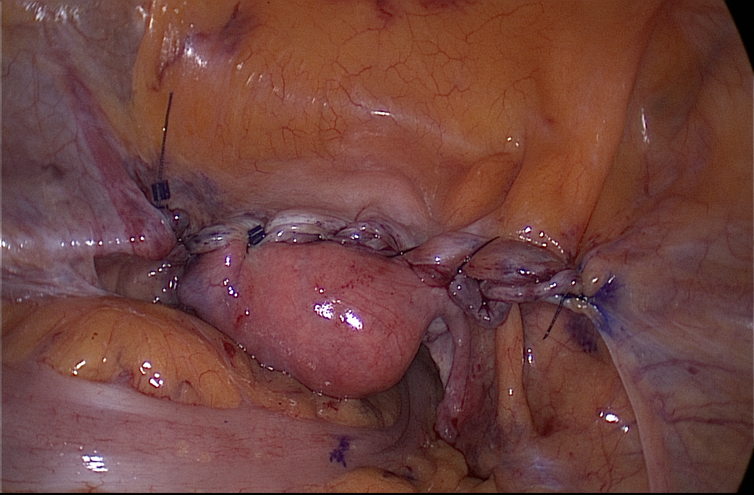

Supplement: Supplementary file 4 — High resolution image (TIFF 678 kb) [file 192_2018_3678_MOESM2_ESM.tiff]
